# Chitosan thermogels for local T lymphocyte delivery for cancer immunotherapy

**DOI:** 10.1186/2051-1426-3-S2-P36

**Published:** 2015-11-04

**Authors:** Anne Monette, Caroline Ceccaldi, Sophie Lerouge, Réjean Lapointe

**Affiliations:** 1 Université de Montréal / Centre de Recherche du Centre Hospitalier de l'Université de Montreal (CRCHUM), Montreal, PQ, Canada; 2 École de technologie supérieure / Centre de Recherche du Centre Hospitalier de l'Université de Montreal (CRCHUM), Montreal, PQ, Canada

## Background

The success of systemic adoptive T cell transfer lies in the capacity of the antigen-experienced cytotoxic T lymphocytes to access and persist within the tumour microenvironment. The mimicking of tertiary lymphoid structures that promote a protective immune response against cancer can be achieved using an injectable biocompatible matrix that releases anti-tumour, proliferating T lymphocytes. Prime candidates for this application are liquid, chitosan-based, biocompatible thermogels which rapidly gelify at physiological temperatures. Therefore, we aimed to fine-tune an injectable chitosan-based thermogel formulation that would provide an environment permitting the three-dimensional proliferation and release of T lymphocytes whose activation state can be influenced by the surrounding conditions. We have developed a novel chitosan formulation that is both cytocompatible and injectable, and which has ideal mechanical properties and porosity for T cell encapsulation and growth. With such promising characteristics, we hypothesize that the injection of these T lymphocytes loaded hydrogels into the tumour microenvironment will provide a means for a continuous delivery of T lymphocytes towards the reprogramming of inflammation mechanisms and the reduction of tumour burden.

## Materials and methods

Novel T cell cytocompatible chitosan thermogels were prepared using combinations of gelling agents. Their rheological properties, mechanical strengths, pH, osmolality, and morphology were evaluated. Three formulations were selected for human T cell encapsulation. Biocompatibility was assessed using live/dead staining and fluorescent microscopy. Thermogel- and supernatant-derived T cells and tumour infiltrating lymphocytes were immunophenotyped over time using flow cytometry.

## Results and conclusions

We have optimized thermogel formulations supporting the encapsulation of T lymphocytes *in vitro*. Use of flow cytometry and microscopy demonstrates which novel thermogel formulation is best suited for cell viability, proliferation, and escape over time, along with the maintenance of T lymphocytes cellular phenotype and an activation status that can be influenced by surrounding conditions. Our injectable three-dimensional T lymphocyte cultures may serve to complement current adoptive cell transfer immunotherapies.

